# Supplementary material for: The medial occipital longitudinal tract supports early stage encoding of visuospatial information
Source: Commun Biol. 2022 Apr 5;5:318. doi: 10.1038/s42003-022-03265-4 (PMC8983765; doi:10.1038/s42003-022-03265-4)
Supplement: Supplementary file 1 — Supplementary Material [file 42003_2022_3265_MOESM1_ESM.pdf]

Supplementary Note 1: Further analysis of the connectivity profiles of the PPA clusters

In our main analysis, we found overall good agreement between the structural and functional connectivity profiles of the posterior and anterior parahippocampal place area (PPA) clusters, but the results were more ambiguous for the lateral cluster. Therefore, we performed further analysis to generate intramodal comparisons for each pair of clusters. To this end, for each pair of clusters, we ran a Spearman rank correlation based on the connectivity values of those two clusters to each vertex of the large ROI encompassing early visual cortex (EVC), the retrosplenial complex (RSC), and medial parietal cortex (MPC). The results of these comparisons are summarised in Supplementary Figure 1 for structural connectivity, and in Supplementary Figure 2 for functional connectivity.

Note that because these comparisons are intramodal, weak correlations bear no substantial value. This is more so the case for functional connectivity due to its lower spatial specificity. This means that the functional connectivity profiles of two clusters should be considered similar only if they are strongly correlated.

The results of the structural connectivity comparisons show no correlations or weak correlations between the observed connectivity profiles of the different PPA clusters (Supplementary Figure 1). Naturally, this should be expected given that the initial hierarchical clustering was based on this information, rendering this a circular question. Comparisons of the functional connectivity values of pairs of clusters corroborate these observations for the posterior and anterior PPA clusters (Supplementary Figure 2). However, the posterior and lateral clusters were indeed strongly correlated in both hemispheres.

To further understand the similarities and differences between the three clusters in an anatomical context, we subtracted the connectivity values of each pair of clusters (within each modality) and plotted the resulting maps on the brain surface (Supplementary Figure 3). As expected, the structural and functional difference maps lend further support to the division between the anterior and posterior PPA clusters: the posterior cluster had a clear preference for EVC, while the anterior cluster showed a clear preference for the RSC/MPC. The comparison of the posterior and lateral clusters revealed that the posterior PPA's structural connectivity to anterior EVC is stronger than that of the lateral PPA, in both hemispheres. Further, although there is almost no difference between the

two clusters based on functional connectivity in the left hemisphere, the posterior cluster is more functionally connected to anterior EVC than the lateral cluster in the right hemisphere (albeit the difference is not very large).

To explain the observed discrepancy between the two modalities, we need to turn to the anatomical properties of the region surrounding the PPA. As functionally defined, the PPA sits in the collateral sulcus, with one portion spanning its medial wall, i.e., the parahippocampal gyrus, and one portion spanning its lateral wall, i.e., the fusiform gyrus. This raises two possible scenarios that may drive the observed functional and structural connectivity profiles. The first scenario relates to the limited ability of fMRI to distinguish the BOLD signal originating from the two walls of a sulcus. fMRI suffers from partial volume effects and mixed signal from neighbouring gyri. It is therefore possible that the PPA's functional localisation encroaches on the lateral wall of the Cos more than the real underlying neuronal populations do. The second possible scenario is that tractography is more capable of visualising connections between EVC and the medial wall of the collateral sulcus. This is especially possible given the known limitations of tractography in reaching deep sulcal locations, an effect known as the 'gyral bias' [1]. In this case, the collateral sulcus may form an artificial boundary for tractography which drives the clustering to show two different zones in the posterior side of the PPA.

It is difficult to determine which of these two scenarios is driving the observed difference between structural and functional connectivity for the lateral PPA cluster, and it is likely that the data is affected by both to some extent. However, the fact that the posterior cluster is more functionally connected to the anterior EVC only in the right hemisphere (Supplementary Figure 3, second column) indicates that there is indeed a functional difference between the two clusters and that they are not identical.

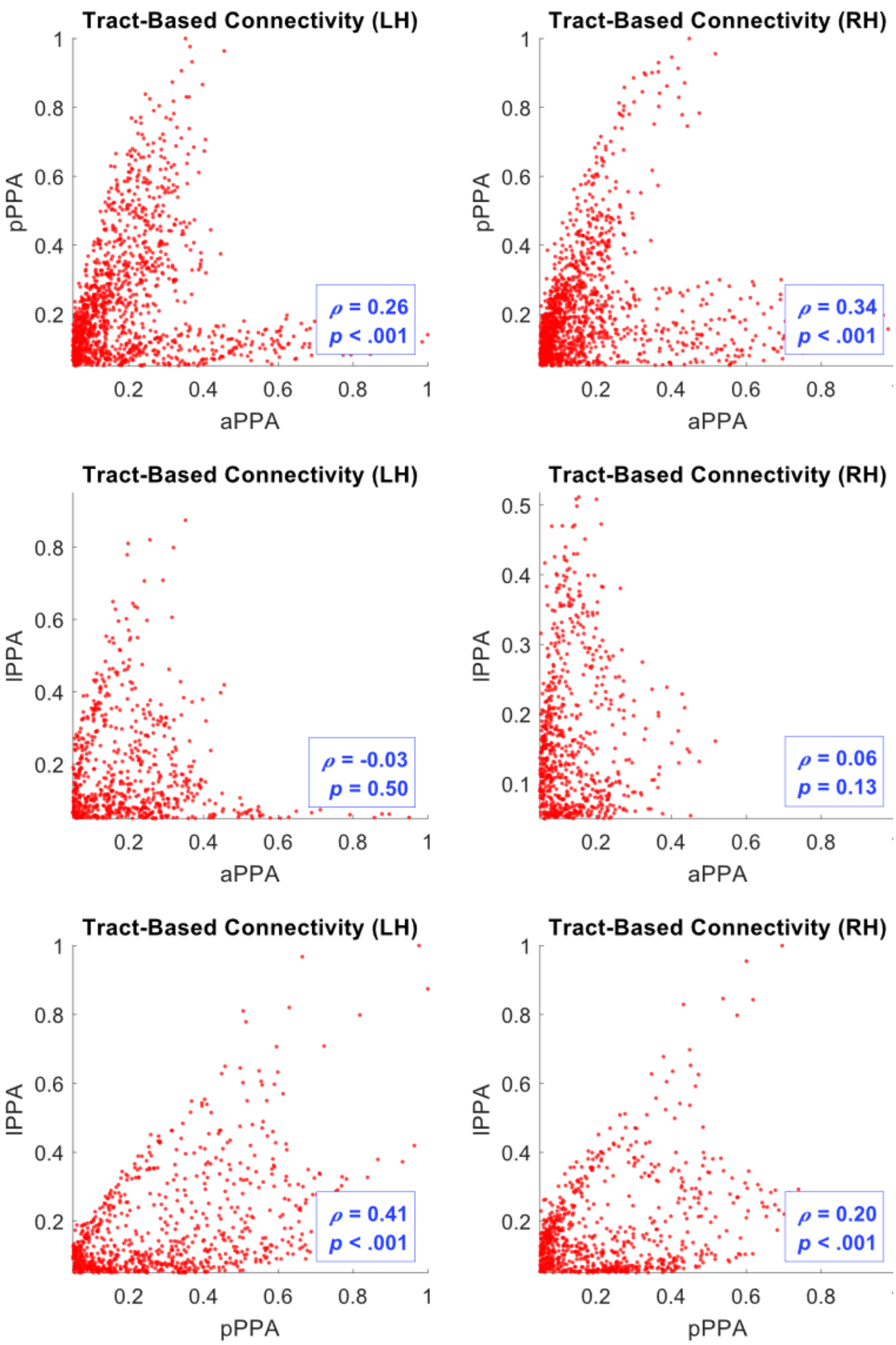

Supplementary Figure 1. Intramodal comparison of the structural connectivity of PPA clusters.

Each chart plots tract-based connectivity values for one PPA cluster against those of another. The text boxes show Spearman rank correlations.

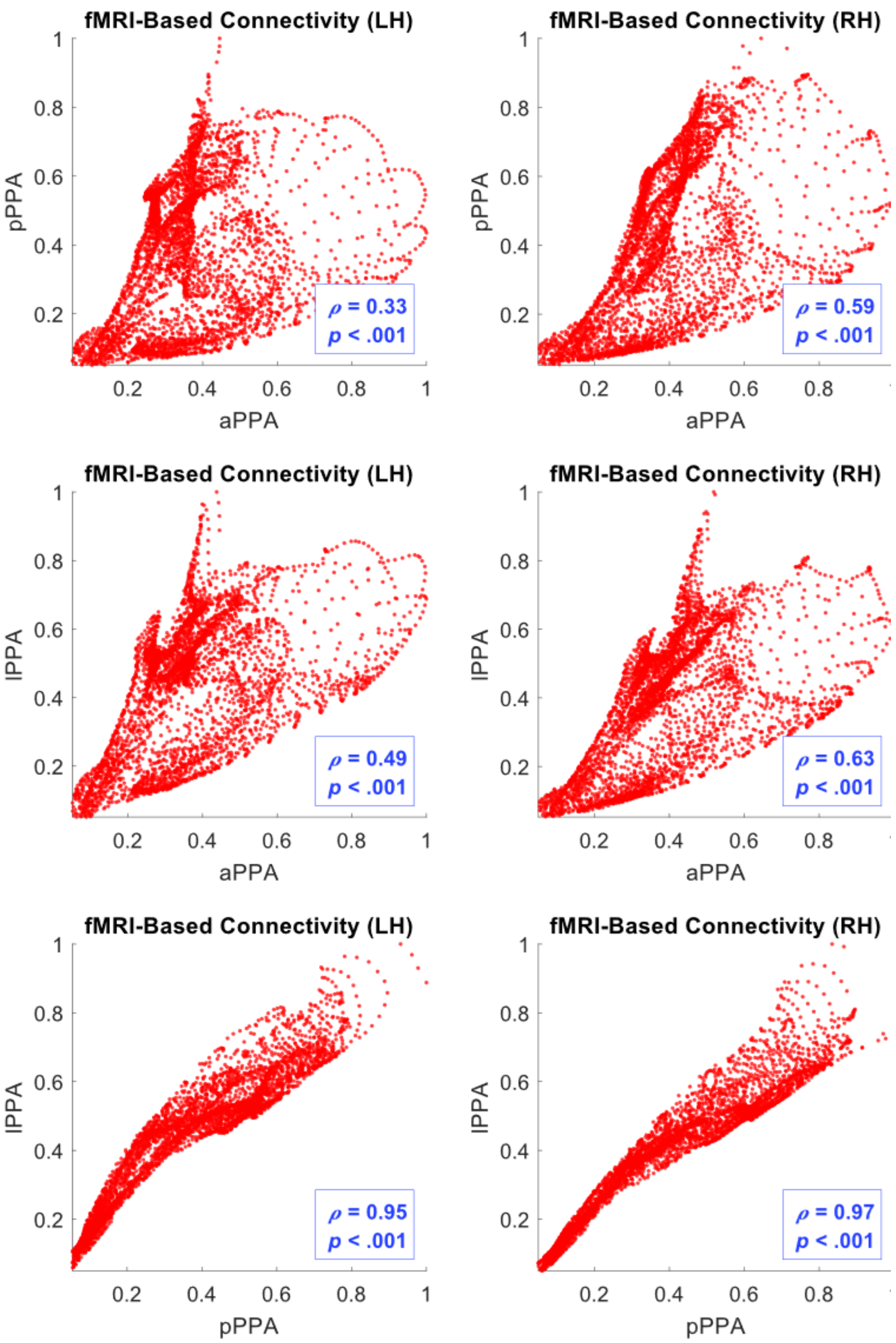

Supplementary Figure 2. Intramodal comparison of the functional connectivity of PPA clusters.

Each chart plots fMRI-based connectivity values for one PPA cluster against those of another. The text boxes show Spearman rank correlations.

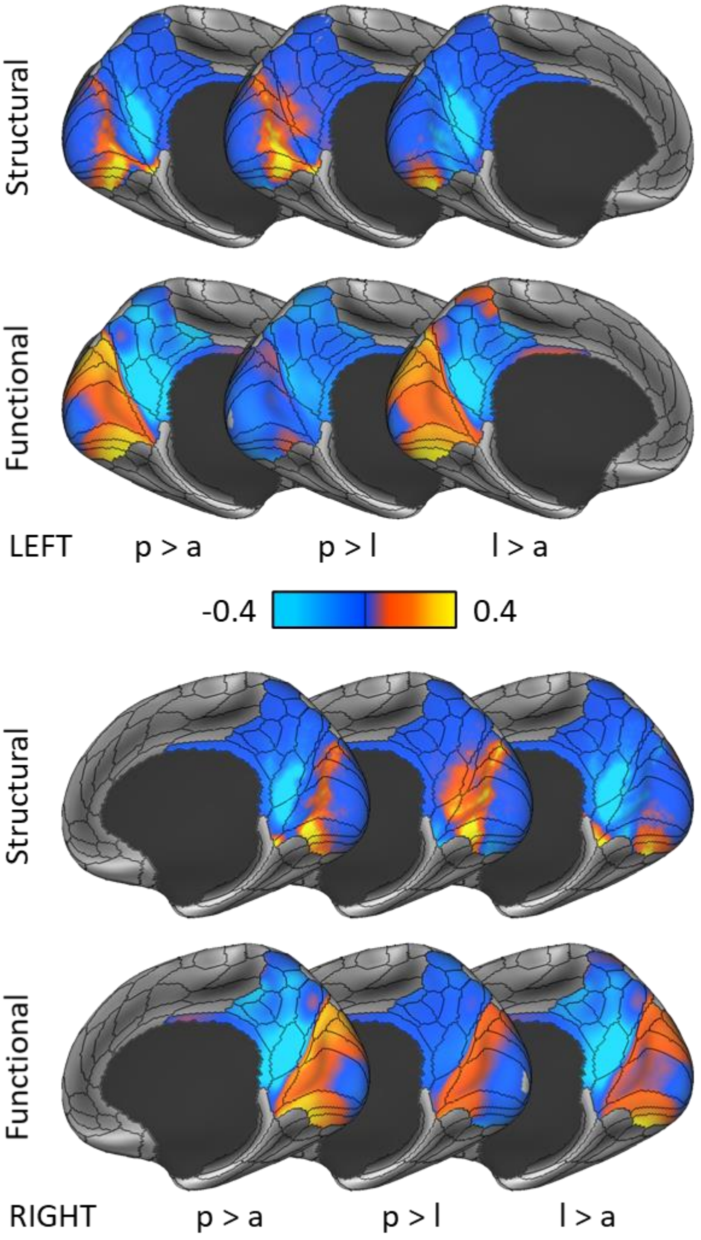

Supplementary Figure 3. Intramodal comparison of the connectivity profiles of each pair of PPA clusters on the brain surface.

To further clarify the similarities and differences between the connectivity profiles of the three PPA clusters, each pair of clusters were additionally compared by direct subtraction. Despite the apparent functional similarity between the posterior and lateral clusters previously found, the posterior cluster still shows higher functional connectivity to anterior EVC in the right hemisphere compared with the lateral cluster. p: pPPA; l: lPPA; a: aPPA. The scale bar indicates the value of the subtracted connectivity strength between the two clusters in question for each panel, e.g., posterior-anterior. For functional connectivity, this corresponds to a difference in the Pearson correlation values. For structural connectivity, this corresponds to the difference in the normalised connectivity value in arbitrary units.

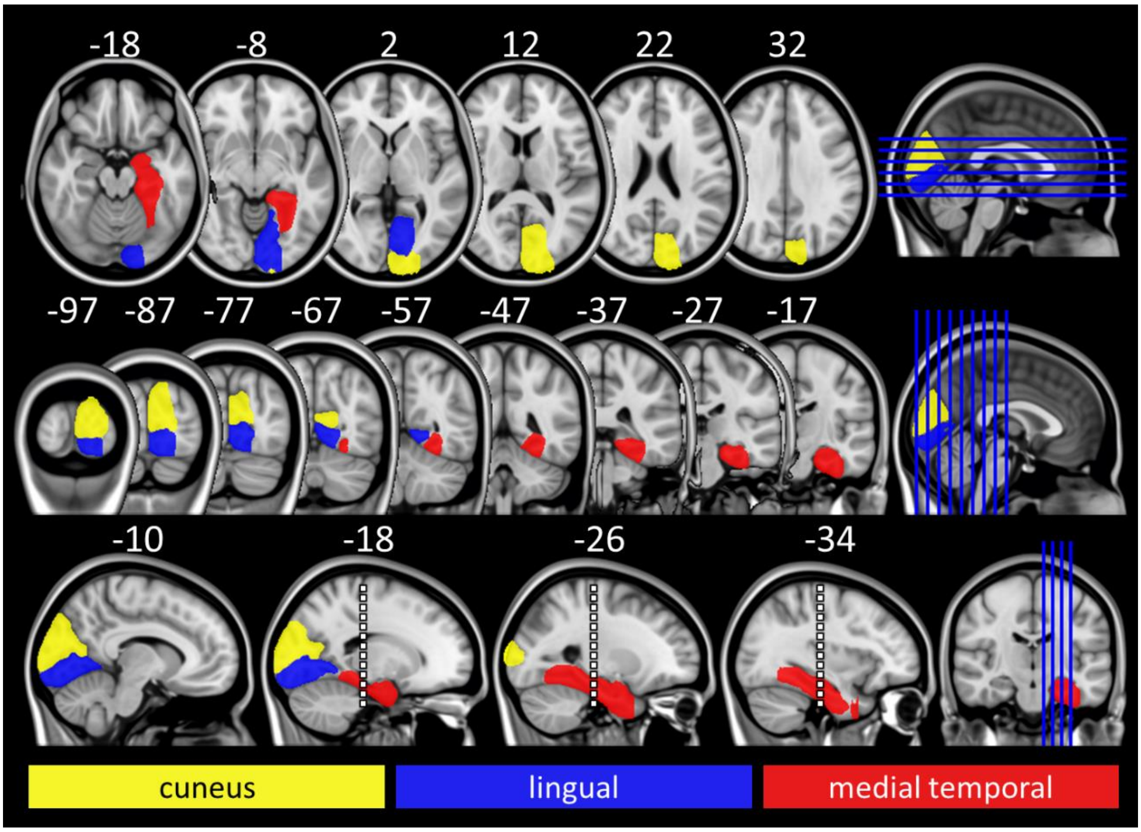

Supplementary Figure 4. Regions of interest used for tractography dissections.

During the creation of the MegaTrack group tractogram, the medial temporal region of interest (ROI) was used to retain streamlines terminating within the medial temporal lobe only. The other ROIs were used during the subsequent virtual dissections to define the medial occipital longitudinal tract (MOLT). The dorsal MOLT was defined by including streamlines terminating in the cuneus and medial temporal ROIs, while the the ventral MOLT was defined using the lingual and medial temporal ROIs. A coronal stopping ROI was added at -26 mm (dashed lines on the sagittal view) to limit the anterior extent of streamlines. Left hemisphere ROIs are displayed here on the MNI152 template and slice numbers indicate MNI coordinates.

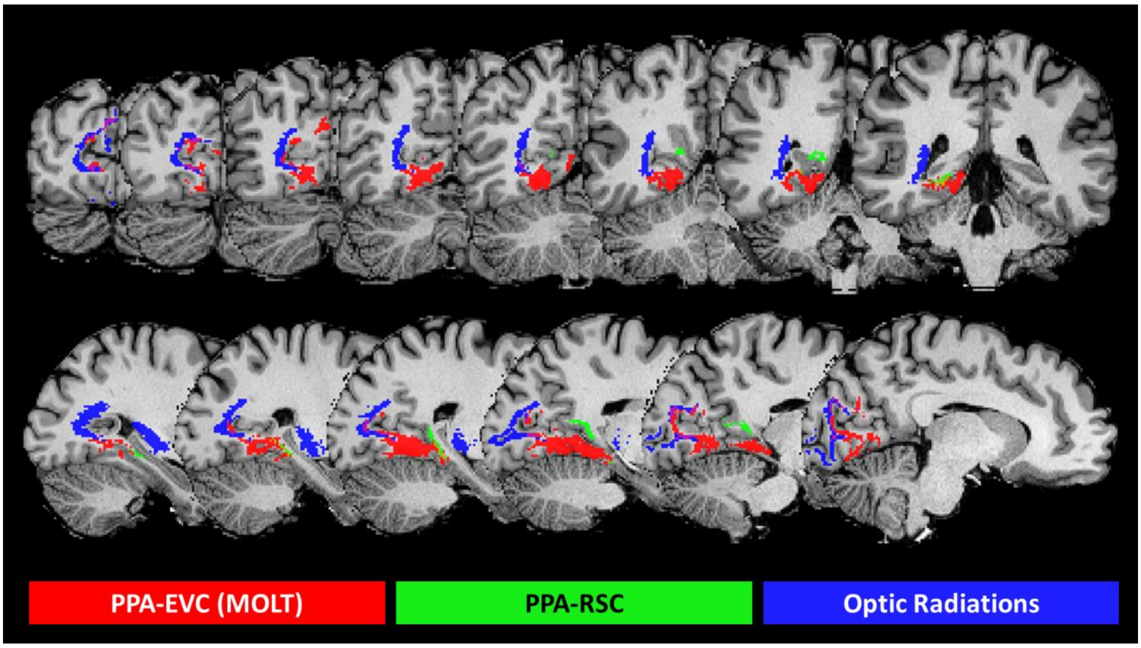

Supplementary Figure 5. Overlays of tract masks displayed for an example subject.

A mask of the medial occipital longitudinal tract (MOLT), which connects the parahippocampal place area (PPA) with early visual cortex (EVC), is overlayed in red on the T1w image of an example subject. To clarify the anatomical location of the MOLT, two additional tracts are displayed as well: the tract connecting the PPA with the retrosplenial complex (RSC) is shown in green, and the optic radiations are shown in blue. The MOLT's trajectory is ventromedial to the occipital horn of the lateral ventricles, which clearly distinguishes it from the optic radiations. The PPA-RSC connections and the MOLT merge anteriorly at the confluence of the posterior cingulate gyrus, posterior parahippocampal gyrus, and anterior lingual gyrus.

Supplementary Table 1. Descriptive statistics of the subcomponents of the medial occipital longitudinal tract (MOLT).

Component	Streamline count	Tract volume (millilitres)	Connected area (mm^2)	HMOA
Dorsal Left	126.84 ± 111.24	3.33 ± 1.76	1360.19 ± 544.86	0.0147 ± 0.0026
Ventral Left	265.28 ± 117.33	7.45 ± 2.20	2817.19 ± 546.70	0.0101 ± 0.0010
Dorsal Right	169.84 ± 125.70	3.74 ± 1.65	1681.94 ± 560.25	0.0149 ± 0.0024
Ventral Right	368.38 ± 176.45	9.24 ± 2.43	3048.01 ± 522.36	0.0106 ± 0.0011

Supplementary Table 2. Statistical comparison of the left and right hemisphere MOLT components.*

Component	Metric	Mean	95% CI	*t*	df	*p*
Cuneus	HMOA	0.01	-0.01, 0.02	1.27	198	.205
	Volume	0.07	0.01, 0.14	3.15	198	.002
	Cortical Area	0.11	0.06, 0.17	6.20	198	< .001
Lingual	HMOA	0.02	0.01, 0.03	6.03	199	< .001
	Volume	0.11	0.08, 0.14	10.98	199	< .001
	Cortical Area	0.04	0.02, 0.06	6.68	199	< .001

* Positive values indicate a right hemisphere lateralisation

Supplementary Table 3. Statistical comparison of the dorsal (cuneus, Cu) and ventral (lingual gyrus, LG) MOLT components.*

Hemisphere	Metric	Mean	95% CI	*t*	df	*p*
Left	HMOA	0.18	0.16, 0.20	29.69	198	< .001
	Volume	-0.41	-0.45, -0.36	-26.66	198	< .001
	Cortical Area	-0.37	-0.40, -0.33	-29.70	198	< .001
Right	HMOA	0.16	-0.45, -0.31	-15.70	199	< .001
	Volume	-0.43	-0.47, -0.39	-32.12	199	< .001
	Cortical Area	-0.30	0.15, 0.18	28.67	199	< .001

* Negative values indicate a dominance of the LG component over the Cu component

Supplementary References

1. Schilling, K. *et al.* Confirmation of a gyral bias in diffusion MRI fiber tractography. *Hum. Brain Mapp.* **39**, 1449–1466 (2018).
