# Supplementary material for: The medial occipital longitudinal tract supports early stage encoding of visuospatial information
Source: Commun Biol. 2022 Apr 5;5:318. doi: 10.1038/s42003-022-03265-4 (PMC8983765; doi:10.1038/s42003-022-03265-4)
Supplement: Supplementary file 2 — Description of Additional Supplementary Files [file 42003_2022_3265_MOESM2_ESM.pdf]

Description of Additional Supplementary Files

File name: Supplementary Data 1
Description: This file contains the y-coordinates and density of projections for each of the EVC and RSC/MPC regions within the MTL. Data from both hemispheres is provided.

File name: Supplementary Data 2
Description: This file contains the variance explained for each of the first 20 principal components identified within the PPA, and the separationspread index for the first a number of clusters ranging from 1 to 25. Data from both hemispheres is provided.

File name: Supplementary Data 3
Description: This file contains the y-coordinates and maximum meta-analytic z-statistic for the terms 'encoding' and 'retrieval' within the PPA. Data from both hemispheres is provided.

File name: Supplementary Data 4
Description: This file contains the original values for each of the 200 subjects, for each metric reported in Figure 6. These values can be used to compute descriptive statistics or to compute the lateralization or vertical bias values reported in the figure according to Equations 1 and 2 in the Methods section.
