# Supplementary material for: The medial occipital longitudinal tract supports early stage encoding of visuospatial information
Source: Commun Biol. 2022 Apr 5;5:318. doi: 10.1038/s42003-022-03265-4 (PMC8983765; doi:10.1038/s42003-022-03265-4)
Supplement: Supplementary file 7 — Nature Reporting Summary [file 42003_2022_3265_MOESM7_ESM.pdf]

nature research

Corresponding author(s): Ahmad Beyh
ahmad.beyh@kcl.ac.uk

Last updated by author(s): Oct 15, 2021

Reporting Summary

Nature Research wishes to improve the reproducibility of the work that we publish. This form provides structure for consistency and transparency in reporting. For further information on Nature Research policies, see our Editorial Policies and the Editorial Policy Checklist.

Statistics

For all statistical analyses, confirm that the following items are present in the figure legend, table legend, main text, or Methods section.

n/a	Confirmed	
☐	☒	The exact sample size (*n*) for each experimental group/condition, given as a discrete number and unit of measurement
☐	☒	A statement on whether measurements were taken from distinct samples or whether the same sample was measured repeatedly
☐	☒	The statistical test(s) used AND whether they are one- or two-sided<br>*Only common tests should be described solely by name; describe more complex techniques in the Methods section.*
☒	☐	A description of all covariates tested
☐	☒	A description of any assumptions or corrections, such as tests of normality and adjustment for multiple comparisons
☐	☒	A full description of the statistical parameters including central tendency (e.g. means) or other basic estimates (e.g. regression coefficient) AND variation (e.g. standard deviation) or associated estimates of uncertainty (e.g. confidence intervals)
☐	☒	For null hypothesis testing, the test statistic (e.g. F, t, r) with confidence intervals, effect sizes, degrees of freedom and P value noted<br>*Give P values as exact values whenever suitable.*
☒	☐	For Bayesian analysis, information on the choice of priors and Markov chain Monte Carlo settings
☒	☐	For hierarchical and complex designs, identification of the appropriate level for tests and full reporting of outcomes
☐	☒	Estimates of effect sizes (e.g. Cohen's *d*, Pearson's *r*), indicating how they were calculated

Our web collection on statistics for biologists contains articles on many of the points above.

Software and code

Policy information about availability of computer code

Data collection	N/A - Secondary data use, cf: Human Connectome Project (https://www.humanconnectome.org).
Data analysis	FSL (RRID:SCR_002823); StarTrack (http://www.mr-startrack.com/); TrackVis (RRID:SCR_004817); MATLAB (RRID:SCR_001622); Connectome Workbench (RRID:SCR_008750); ANTS - Advanced Normalization ToolS (RRID:SCR_004757); NeuroSynth (RRID:SCR_006798).

For manuscripts utilizing custom algorithms or software that are central to the research but not yet described in published literature, software must be made available to editors and reviewers. We strongly encourage code deposition in a community repository (e.g. GitHub). See the Nature Research guidelines for submitting code & software for further information.

Data

Policy information about availability of data

All manuscripts must include a data availability statement. This statement should provide the following information, where applicable:

- Accession codes, unique identifiers, or web links for publicly available datasets
- A list of figures that have associated raw data
- A description of any restrictions on data availability

The data used for this study are freely available from the Human Connectome Project (https://www.humanconnectome.org/).

Field-specific reporting

Please select the one below that is the best fit for your research. If you are not sure, read the appropriate sections before making your selection.

☐ Life sciences ☒ Behavioural & social sciences ☐ Ecological, evolutionary & environmental sciences

For a reference copy of the document with all sections, see nature.com/documents/nr-reporting-summary-flat.pdf

Behavioural & social sciences study design

All studies must disclose on these points even when the disclosure is negative.

Study description	Quantitative data-driven analysis in a large control cohort.
Research sample	Large cohort (N = 200) from the Human Connectome Project (HCP), 100 F/100 M, mean age 29.16 ± 3.73 years.
Sampling strategy	The control cohort was chosen to ensure a good balance between male and female participants (100 F, 100 M) and to minimise confounding effects from handedness (all right-handed). The sample choice explicitly excluded twins.
Data collection	Secondary data use, cf: Human Connectome Project (https://www.humanconnectome.org).
Timing	N/A.
Data exclusions	No data were excluded from the chosen HCP sample.
Non-participation	N/A.
Randomization	N/A.

Reporting for specific materials, systems and methods

We require information from authors about some types of materials, experimental systems and methods used in many studies. Here, indicate whether each material, system or method listed is relevant to your study. If you are not sure if a list item applies to your research, read the appropriate section before selecting a response.

Materials & experimental systems

n/a	Involved in the study	
☒	☐	Antibodies
☒	☐	Eukaryotic cell lines
☒	☐	Palaeontology and archaeology
☒	☐	Animals and other organisms
☐	☒	Human research participants
☒	☐	Clinical data
☒	☐	Dual use research of concern

Methods

n/a	Involved in the study	
☒	☐	ChIP-seq
☒	☐	Flow cytometry
☐	☒	MRI-based neuroimaging

Human research participants

Policy information about studies involving human research participants

Population characteristics	Data from 200 healthy participants (100 females; age = 29.16 ± 3.73 years) were obtained from the Human Connectome Project (https://www.humanconnectome.org). All subjects were right-handed as determined by a score of 50 and above on the Edinburgh handedness questionnaire (Oldfield, 1971).
Recruitment	See above.
Ethics oversight	Secondary data use, cf: Human Connectome Project (https://www.humanconnectome.org).

Note that full information on the approval of the study protocol must also be provided in the manuscript.

Magnetic resonance imaging

Experimental design

Design type	Mean resting-state fMRI data.
Design specifications	N/A.
Behavioral performance measures	N/A.

Acquisition

Imaging type(s)	Structural, functional, diffusion.
Field strength	3
Sequence & imaging parameters	HCP diffusion MRI data were acquired on a 3 T Siemens “Connectome Skyra” using a spin-echo EPI sequence (TR = 5520 ms; TE = 89.5 ms; matrix of 168x144; 111 slices with a thickness of 1.25 mm; isotropic voxels of 1.25 mm; multiband factor = 3). Three diffusion-weighted shells were acquired (b = 1000, 2000 and 3000 s/mm2) with two opposite phase-encoding directions each (L>>R and R>>L). Each shell consisted of 90 diffusion-weighted directions and six interleaved non-diffusion-weighted volumes. For this study, only data from the b = 2000 s/mm2 shell were used.
Area of acquisition	Brain.

Diffusion MRI [X] Used [ ] Not used

Parameters: b = 2000 s/mm2; 90 directions.

Preprocessing

Preprocessing software	Secondary data use, cf: HCP minimal preprocessing pipelines (Glasser et al., NeuroImage, 2013).
Normalization	Non-linear normalisation using ANTs based on anisotropic power maps derived from the diffusion data.
Normalization template	MNI152.
Noise and artifact removal	Secondary data use, cf: HCP minimal preprocessing pipelines (Glasser et al., NeuroImage, 2013).
Volume censoring	Secondary data use, cf: HCP minimal preprocessing pipelines (Glasser et al., NeuroImage, 2013).

Statistical modeling & inference

Model type and settings	Secondary data use, cf: HCP minimal preprocessing pipelines (Glasser et al., NeuroImage, 2013).
Effect(s) tested	Secondary data use, cf: HCP minimal preprocessing pipelines (Glasser et al., NeuroImage, 2013).

Specify type of analysis: [X] Whole brain [ ] ROI-based [ ] Both

Statistic type for inference (See Eklund et al. 2016)	Secondary data use, cf: HCP minimal preprocessing pipelines (Glasser et al., NeuroImage, 2013).
Correction	Secondary data use, cf: HCP minimal preprocessing pipelines (Glasser et al., NeuroImage, 2013).

Models & analysis

n/a	Involved in the study
[ ]	[X] Functional and/or effective connectivity
[X]	[ ] Graph analysis
[X]	[ ] Multivariate modeling or predictive analysis

Functional and/or effective connectivity	Pearson's R (HCP 1200 Subject Release Reference Manual - secondary data use).
